# Transgressive phenotypes and evidence of weak postzygotic isolation in F1 hybrids between closely related capuchino seedeaters

**DOI:** 10.1371/journal.pone.0199113

**Published:** 2018-06-14

**Authors:** Leonardo Campagna, Pablo Rodriguez, José Carlos Mazzulla

**Affiliations:** 1 Department of Ecology and Evolutionary Biology, Cornell University, Ithaca, NY, United States of America; 2 Fuller Evolutionary Biology Program, Cornell Lab of Ornithology, Cornell University, Ithaca, NY, United States of America; 3 Federación Ornitológica Argentina, Virrey Liniers, Ciudad Autónoma de Buenos Aires, Argentina; 4 Asociación Canaricultores Roller del Uruguay, Montevideo, Uruguay; University of Arkansas, UNITED STATES

## Abstract

Postzygotic reproductive isolation may become strong only once the process of speciation is in its advanced stages. For taxa in the early stages of speciation, prezygotic reproductive isolation barriers may play a predominant role in maintaining species boundaries. Here, we study the recent capuchino seedeater biological radiation, a group of highly sympatric species from the genus *Sporophila* that have diversified during the Pleistocene in Neotropical grasslands. Capuchinos can be diagnosed by adult male coloration patterns and song, two sets of characters known to contribute to pre-mating reproductive isolation. However, it remains unknown whether potzygotic incompatibilities contribute to maintaining species limits in this group. Here we use existing breeding records from captive individuals to test for patterns consistent with F1 inviability. We compare hatching success, fledging success, and the sex ratio at adulthood between conspecific and hybrid capuchino pairs. We observed a trend towards lower numbers of the heterogametic sex among adult hybrids, consistent Haldane's rule, but this was supported by only one of our statistical tests. Our study is the first to document hybrid male capuchino phenotypes based on known crosses. We observed phenotypes that were similar or intermediate to those of the parental species, as well as novel plumage patterns that have not been described in the wild. One cross produced a plumage pattern that has been observed at low frequencies in natural populations. We discuss the implications of our results for understanding the relative importance of the mechanisms of reproductive isolation in capuchino seedeaters.

## Introduction

Postzygotic reproductive isolation barriers among avian species have been shown to develop over millions of years, generally becoming progressively stronger as species diverge [1–4]. A few common patterns have been found in the way these postzygotic incompatibilities accumulate across different avian groups. One such pattern is Haldane’s rule [5], which predicts that in hybrids, the heterogametic sex (ZW females in birds), will be the first to suffer infertility or inviability. Infertility, in particular, has been found to arise before inviability [1]. One explanation for the patterns predicted by Haldane’s rule is that selection can act on favorable recessive mutations when they are hemizygous in the heterogametic sex [6, 7]. This leads to increased divergence in Z-linked loci between species, and some of these loci may interact epistatically with autosomal loci. These divergent Z-linked loci may have detrimental effects in hybrids; for example, they may not be compatible with alleles present at an autosomal locus in a different species. These incompatibilities between recessive Z-linked loci from one species and autosomal loci from a different species will arise first in those hybrids from the heterogametic sex, where the Z-linked recessive loci are exposed to selection [6, 7]. A second common pattern is that inviability is higher in F2 hybrids and backcrosses in comparison to F1 hybrids [2, 4] due to the increasing number and severity of "Bateson-Dobzhansky-Muller" incompatibilities in later generation hybrids (see [8]). These patterns of postzygotic incompatibilities have been observed by studying hybrid crosses across a wide spectrum of divergence, and generally show that it is likely that strong postzygotic reproductive isolation takes longer to arise than average speciation times [1]. This suggests that premating isolation mechanisms may be especially important in maintaining avian reproductive isolation [1, 3], at least in the early stages of speciation.

**Fig 1:**
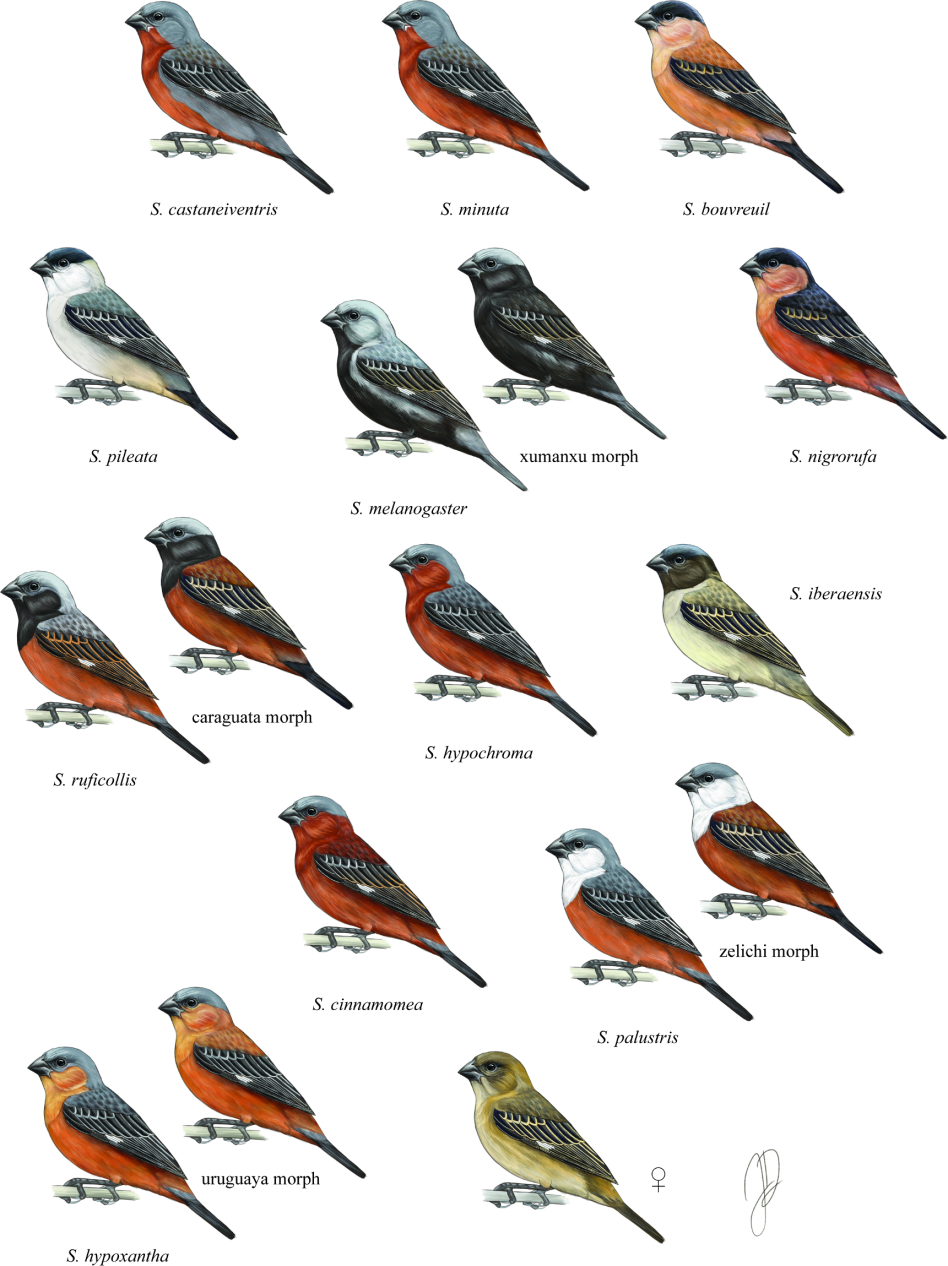
The capuchino seedeaters. Plumage diversity in the eleven species of capuchinos, the four alternative coloration morphs, and the recently described *S*. *iberaensis*. Illustrations by Jillian Ditner.

Instances of recent and rapid speciation may help shed light on the mechanisms that contribute to reproductive isolation. The elevated pace of speciation in such cases could be related to a fast accumulation of postzygotic incompatibilities, disproportionally strong levels of premating isolation, or both. Here we explore the fitness consequences of crosses among capuchino seedeaters in the genus *Sporophila*, a group of 11 species that evolved rapidly throughout the Pleistocene in the Neotropics [9, 10]. Nine of these species are currently highly sympatric and likely diverged from a common ancestor within the last million years. The males of these different species vary in their adult reproductive plumage and in their songs [11] (Fig 1), traits that are widely implicated in generating pre-mating reproductive isolation in birds [3]. Male capuchinos distinguish between conspescific and heterospecific song in sympatry, responding more aggressively to conspecifics during playback experiments [12], suggesting that differences in song convey species information. Capuchinos are sexually dimorphic and, in contrast to males, females are pale brown and phenotypically indistinguishable across the different species [11]. Outside of the reproductive season males molt into “eclipse” plumage, resembling females [13, 14]. Despite this phenotypic variation, capuchinos are genetically very similar, differing in only a small number of regions across their genome [15, 16]. These divergent regions are enriched in genes from the melanogenesis pathway [16], presumably the genes responsible for the variation in male coloration patterning.

In other similarly closely related or recent pairs of species, hybrids and backcrosses can be found in nature, albeit in geographically restricted areas and/or at low frequency (e.g., Hooded and Carrion crows, [17]; Golden-winged and Blue-winged warblers, [18]; munias, [19]). In capuchino seedeaters, however, it is not clear how prevalent hybridization is in the wild, or even if it occurs at all, and therefore information on postzygotic isolation is lacking. This is partially because only adult males in reproductive plumage can be confidently identified to species. Therefore, the identification of putative hybrids is restricted to cases of adult males showing abnormal reproductive plumages. Despite the limitation of only being able to detect putative hybrids in a single sex and age class, a number of unusual coloration patterns have been described in four capuchino seedeater species: *Sporophila hypoxantha* has an alternative coloration morph known as *uruguaya* [20], *S*. *melanogaster* presents the *xumanxu* morph [21], *S*. *ruficollis* the *caraguata* morph [22] and *S*. *palustris* the *zelichi* morph (formerly recognized as *S*. *zelichi*, [23]—see Fig 1 for representations of both morphs). In these cases the alternative morphs are rare in the wild (sometimes only a handful of individuals have been observed) and are considered intraspecific coloration morphs of the species whose diagnostic song they sing. The putative species *S*. *iberaensis* (Fig 1), described by Di Giacomo and Kopuchian [24] and named after the Esteros del Iberá wetlands in Argentina, is an exception to this pattern. Although a quantitative analysis of song differences and genetic studies are still lacking, this form represents a novel phenotype that is both locally common and for which males sing a diagnostic song. Whether these color morphs represent segregating polymorphisms within particular species or are the result of hybridization is difficult to determine. The absence of intermediate song phenotypes has been taken as evidence against their hybrid origin [20, 22, 23]. However, hybrids would not be expected to sing intermediate songs if this cultural trait (in oscine passerines) were learned from the male parent in a hybrid cross, or if these hybrids were the results of extra-pair mating [25]. Sexual signals with different modes of inheritance (e.g., cultural and genetic) can be decoupled in areas where gene flow occurs among incipient species [26].

*Sporophila* seedeaters, including capuchinos, are common cage birds in South America [13]. Most of these captive birds are wild-caught yet can be bred in captivity. In some cases, the trapping pressure from the pet-trade is sufficiently strong to represent a serious conservation threat [27, 28]. Here we take advantage of breeding records (both conspecific and hybrid crosses) originally compiled by hobbyist aviculturists during efforts to establish lines of captive-born capuchinos that would alleviate the trapping pressure on wild birds from the illegal pet trade. Although these records were not generated as part of a planned study, we can use them post hoc to ask: What novel capuchino phenotypes can be produced via hybridization? Do any of these phenotypes coincide with those of the described intra-specific color morphs that exist in the wild? Is there evidence of reduced viability in F1 hybrids?

## Materials and methods

### Animal husbandry

The individual birds used in this study were either born in the wild or raised from birds kept in captivity for up to two generations. All individuals were identified by unique combinations of bird bands and were kept in avian breeding facilities. Breeding data were compiled over the course of 11 years (2006–2016). Details on the conditions in which capuchino seedeaters can be bred in captivity were published elsewhere (see [29, 30]). During non-reproductive periods, birds were housed in groups in large cages (approximately 150 cm in length and 30 cm in width and height). Groups consisted of 10–15 individuals segregated by sex into different cages. The reproductive period begins in September/October and lasts until March. When females showed evidence of reproductive activity (e.g., while adopting copulatory poses in response to male song) they were moved to smaller individual cages (approximately 60 cm long, 30 cm wide and 30 cm high). Males were also moved to individual cages once they began to sing and show reproductive plumage. All cages had perches for birds and were cleaned regularly. The cages housing females contained a wire structure in the shape of a bowl nest with its interior covered in string. Nesting material (e.g., dry grass) was provided for females to line the nest. Birds were fed a mix of seeds, fruits and vegetables, with occasional vitamin supplementation.

### Breeding experiments

The cages housing the individuals of each sex were placed adjacently and initially separated by physical and visual barriers. The contact with singing males stimulates females to build nests. The visual barrier was removed once the nest building began. If a female responded to the male’s presence (e.g., by soliciting copulation), the physical barrier between cages was removed. Generally, the male entered the female’s cage and copulated. The process was repeated 2–3 times a day for 2 or 3 days until the female began to reject the male. Females incubated the eggs and subsequently fed the chicks alone. Females lay 2–3 eggs, incubate them for 11 days, and 10–12 days after hatching, the chicks fledge. The chicks are fed by the female until up to 35–40 days of age, after which they were removed and placed in the large communal cages. In captivity, each female can have three or four nesting attempts per season.

### Comparisons between conspecific and hybrid pairs

Male birds were identified to species by their diagnostic reproductive plumage. Females were identified using information of the species present in the locality of origin and by the diagnostic plumage coloration of their sons. In captivity older females may also develop some characteristics of the male plumage of their species [29]. A total of 60 mating experiments were performed, involving seven of the 11 capuchino species (see Table 1 and S1 Table); 20 crosses were interspecific (i.e., hybridization experiments). All the species involved in the hybrid crosses can occur sympatrically in the wild, although their ranges are not always completely overlapping. We only considered F1 hybrid crosses, although a few F1 hybrids were subsequently backcrossed or crossed to other species in some cases, and we present those results in Table 2. The four capuchino species that were not included in this study were *S*. *castaneiventris*, *S*. *bouvreuil*, *S*. *melanogaster*, and *S*. *nigrorufa*. We included males of the alternative morphs from S. *ruficollis* (*caraguata*), S. *hypoxantha* (*uruguaya*) and S. *palustris* (*zelichi*), but not from *S*. *melanogaster* (*xumanxu*). For each pair, we recorded the number of eggs laid, the number of eggs hatched, the number of chicks that fledged (between 35 and 40 days of age), and their sex when possible. The data were combined when a pair had more than one nesting attempt in a year. Sex was determined by the development of adult reproductive plumage, therefore remaining unknown when individuals died before reaching this stage. Some individuals show a few feathers with adult coloration between six and eight months of age but develop their full reproductive plumage only when two or three years old [29]. From these data we derived three variables: hatching success, fledging success and sex ratio of adults. We defined hatching success as the proportion of laid eggs that hatched. Similarly, fledging success was the proportion of hatched eggs that produced chicks that fledged. The sex ratio of adults was calculated as the number of adult males divided by the total number of adults for which information of sex was available (M/(F+M)).

**Table 1:** Details for the 60 breeding experiments performed in this study pooled by the species involved in the pair. For each pair the table specifies the cross type (hybrid or conspecific), total number of eggs laid, total number of eggs hatched, total number of chicks reaching adulthood and their sex when known.

Sp. father	Sp. mother	Type of pair	Number of matings	#Eggs laid	#Eggs hatched	#Adult total	#Adult F1 females	#Adult F1 males	#Adult unknown sex
*S*. *minuta*	*S*. *minuta*	Conspecific	1	4	2	1	1	0	0
*S*. *pileata*	*S*. *pileata*	Conspecific	3	11	4	2	2	0	0
*S*. *cinnamomea*	*S*. *cinnamomea*	Conspecific	14	55	26	8	3	3	2
*S*. *ruficollis*	*S*. *ruficollis*	Conspecific	10	37	19	10	4	3	3
*S*. *palustris*	*S*. *palustris*	Conspecific	10	29	15	9	3	4	2
*S*. *hypochroma*	*S*. *hypochroma*	Conspecific	2	4	1	0	0	0	0
*S*. *palustris* (*zelichi*)	*S*. *hypoxantha*	Hybrid	1	8	6	4	2	2	0
*S*. *palustris*	*S*. *hypoxantha*	Hybrid	1	4	3	2	0	2	0
*S*. *pileata*	*S*. *hypochroma*	Hybrid	1	5	5	3	2	1	0
*S*. *cinnamomea*	*S*. *ruficollis*	Hybrid	1	2	2	1	0	1	0
*S*. *ruficollis*	*S*. *palustris*	Hybrid	1	2	1	1	1	0	0
*S*. *ruficollis* (*caraguata*)^1^	*S*. *ruficollis*	Hybrid	3	7	4	0	0	0	0
*S*. *hypoxantha* (*uruguaya*)	*S*. *cinnamomea*	Hybrid	1	4	2	0	0	0	0
*S*. *palustris*	*S*. *cinnamomea*	Hybrid	7	21	14	6	0	1	5
*S*. *ruficollis*	*S*. *cinnamomea*	Hybrid	4	11	7	3	0	1	2

^1^The *caraguata* morph belongs to *S*. *ruficollis* but was categorized as a hybrid cross because it is phenotypically distinct from the most common male coloration pattern in that species.

**Table 2:** Adult F1 hybrids. The 13 adult F1 hybrids that reached sexual maturity, only four of which were crossed to other species. Three females showed some signs of sterility and one male was fertile.

Hybrid	Sex	Fertility	Crossed to species
*S*. *palustris* (*zelichi*) x *S*. *hypoxantha*	Male	-	-
*S*. *palustris* (*zelichi*) x *S*. *hypoxantha*	Male	-	-
*S*. *palustris* (*zelichi*) x *S*. *hypoxantha*	Female	-	-
*S*. *palustris* (*zelichi*) x *S*. *hypoxantha*	Female	-	-
*S*. *palustris* x *S*. *hypoxantha*	Male	-	-
*S*. *palustris* x *S*. *hypoxantha*	Male	-	-
*S*. *pileata* x *S*. *hypochroma*	Male	-	-
*S*. *pileata* x *S*. *hypochroma*	Female	Sterile^1^	*S*. *cinnamomea*
*S*. *pileata* x *S*. *hypochroma*	Female	Fertile?^2^	*S*. *cinnamomea*
*S*. *cinnamomea* x *S*. *ruficollis*	Male	Fertile	*S*. *ruficollis*
*S*. *ruficollis* x *S*. *palustris*	Female	Sterile^3^	*S*. *ruficollis*
*S*. *palustris* x *S*. *cinnamomea*	Male	-	-
*S*. *ruficollis* x *S*. *cinnamomea*	Male	-	-

^1^Three breeding seasons.

^2^Produced one egg that hatched out of 6 in the third breeding season.

^3^Two breeding seasons (one at age 2 and one at age 4). Laid three eggs with no embryo.

The data were analyzed in two different ways: 1) using the specific pair of individuals as the sampling unit, totaling 60 different pairs (S1 Table); and 2) pooling the raw data (e.g., number of eggs laid) from all pairs of individuals involving the same combination of species (or conspecific matings), totaling 15 unique combinations or same species pairs (Table 1). Once the raw data were pooled we derived the three response variables for each group. For hybrid crosses, data were pooled by the species involved in the crosses, considering directionality separately (e.g., male *S*. *ruficollis* x female *S*. *cinnamomea* was considered separately from male *S*. *cinnamomea* and female *S*. *ruficollis*). Intra-specific color morphs were also considered separately from individuals of the same species that had the most common phenotype (e.g., *S*. *palustris* was considered separately from the *zelichi* morph of *S*. *palustris*). For each variable, we assessed statistical significance when comparing hybrid and conspecific matings using a randomization test. First, we calculated the difference between the means for the observed data. We tested the null hypothesis of no difference between these means (i.e., hybrid–conspecific = 0) by randomizing the “type of cross” label (hybrid or conspecific) 10,000 times while maintaining the group size constant. The null distribution was generated by calculating the difference between the means after each randomization, and the proportion of randomizations that produced a difference in means that was more extreme than the observed one was used as the p-value (multiplied by two for two-tailed tests).

## Results

We obtained data from a total of 60 breeding pairs, 40 of which comprised individuals from the same species (hereafter “conspecific” pairs), involving six different species with between one and 14 matings per species (Table 1). The remaining 20 breeding pairs involved heterospecific crosses (hereafter “hybrid” pairs), comprising nine unique combinations of species and between one and seven crosses per combination (Table 1). The number of breeding pairs was higher for conspecific pairs compared to hybrid crosses (40 vs. 20 pairs), and this was reflected in the total number of eggs that were laid (140 vs. 64; Table 1). Twenty F1 hybrids (out of 44 that hatched) lived long enough to fledge (between 35 and 40 days of age), and for 13 of those (8 males and 5 females) sex could be determined (Table 1). The remaining 7 F1 hybrids died before reaching sexual maturity. Four of the 13 F1 hybrids were involved in subsequent crosses (three females and a male; Table 2). The male was fertile, while the females showed signs of infertility: two females laid eggs that did not hatch on multiple nesting attempts, while the third female laid clutches during three breeding seasons and a single egg hatched in her third year. We note that two of these F1 females were crossed with a male of a third species (i.e., not one of the parental species), possibly confounding our ability to evaluate their fertility.

**Fig 2:**
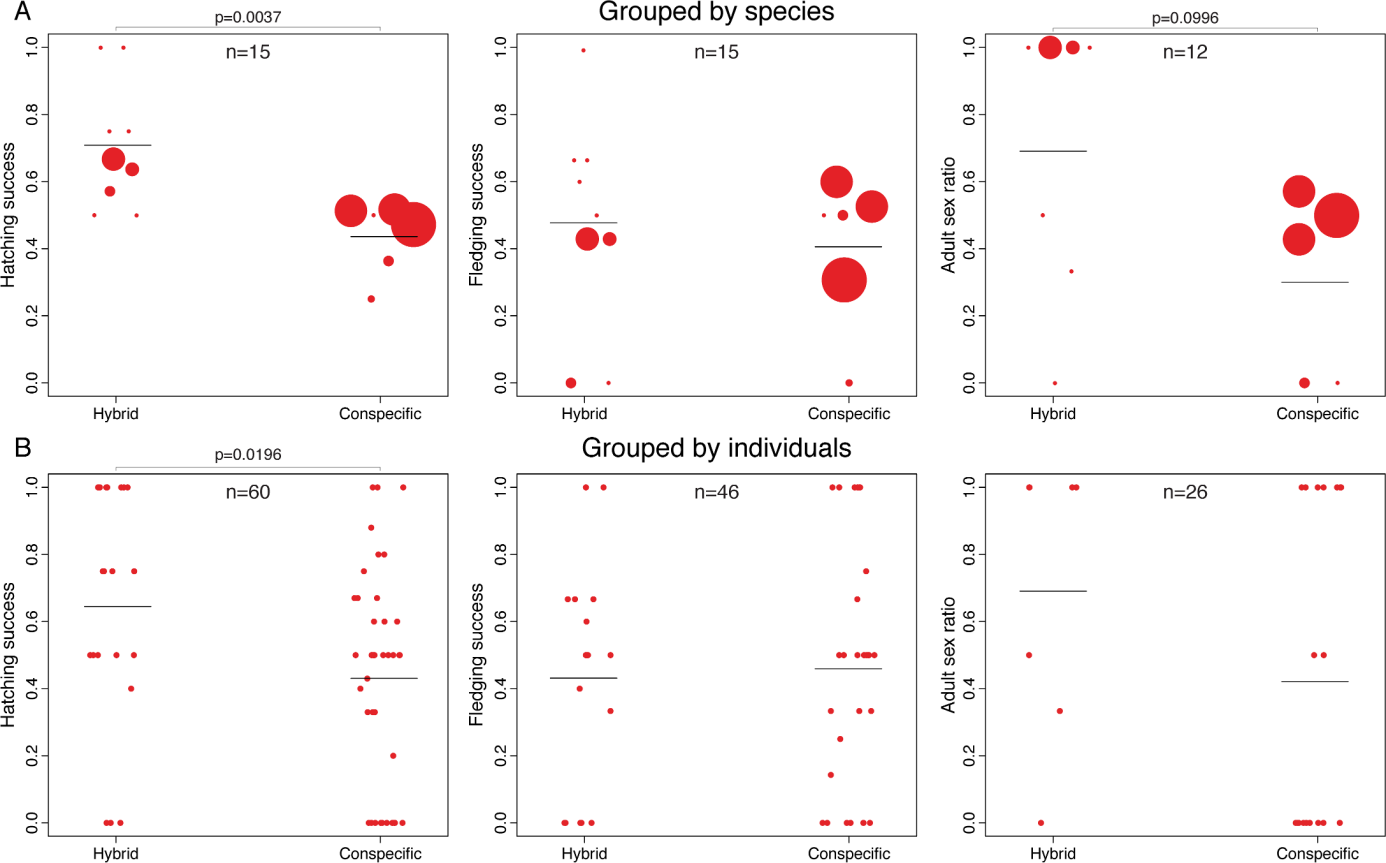
Viability of F1 hybrids. Hatching success, fledging success and adult sex ratio (Males/Males + Females) compared between hybrid and conspecific pairs. Sample sizes vary for the different panels because either the individuals that resulted from the crosses did not reach adulthood or sex could not be determined for all adults. Horizontal lines indicate mean values for each type of cross. All tests are two-tailed. A) Results grouping the data by species (the size of the points is scaled to the number of crosses performed for that combination of species; see Table 1). B) Results grouping the data by pairs of individuals (see methods).

On average 54% of eggs hatched. However, the hatching success was significantly higher for hybrid crosses than for conspecific ones (71% hybrid vs. 44% conspecific, p = 0.0307; Fig 2A). Approximately 45% of the eggs that hatched survived to fledge, and fledging success did not differ significantly between the two types of pairs (Fig 2A). These results were qualitatively similar when analyses were based on individual pairs instead of being grouped by species or type of cross (Fig 2A cf. Fig 2B). The sex ratio of adults, defined as the number of males divided by the total number of individuals for which sex could be determined, was significantly higher in hybrids than in conspecific matings only when we conducted a one-tailed test and pooled the data by species/type of cross (69% hybrid vs. 30% conspecific, one tailed p = 0.0498, two tailed p = 0.0996; Fig 2A). This trend towards an excess of males in the hybrid crosses was not statistically significant when the data were pooled by pairs of individuals (69% hybrid vs. 42% conspecific; Fig 2B).

**Fig 3:**
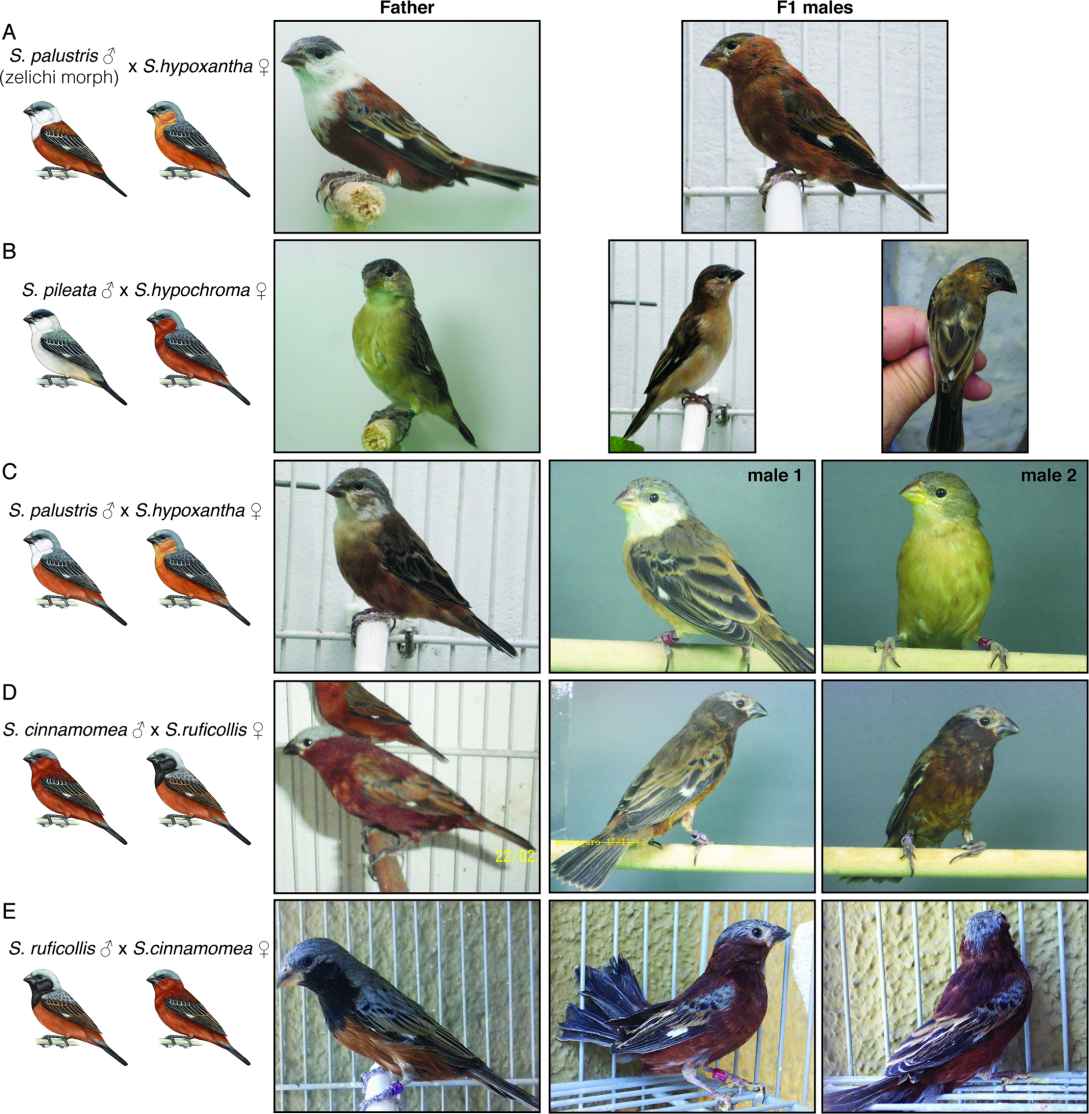
Transgressive phenotypes in F1 capuchino hybrid males. Of the eight adult hybrid males obtained six lived long enough to develop adult reproductive plumage. The parental species and their sex is indicated in the left column, together with the characteristic plumage pattern of the males of that species. The middle column shows pictures of the fathers, while the column on the right contain the pictures of the adult male F1 hybrids. Note that panel C shows two sibling F1 hybrid males.

A total of 8 adult F1 males lived to adulthood, 6 of which survived to develop full reproductive plumage (Fig 3). We compared the adult plumage patterns of these 6 F1 hybrids to that of their fathers and the male phenotype of their mother’s species. In most cases we observed transgressive phenotypes in the adult F1 males; i.e., with coloration and/or patterning that was different from that of the parental species (Fig 3). Fig 3A shows the result from the cross of a *S*. *palustris* (*zelichi* morph) male and a *S*. *hypoxantha* female. This F1 hybrid is phenotypically similar to the low frequency alternative *uruguaya* morph that has been described for *S*. *hypoxantha* (Fig 1). The cross of a regular *S*. *palustris* male to a *S*. *hypoxantha* female resulted in different F1 phenotypes (Fig 3A c.f. Fig 3C), suggesting that there are genetic differences between the regular and *zelichi* morphs of *S*. *palustris*. Fig 3C shows the only case in which we could compare two sibling F1 males, only one of which showed a phenotype similar to that of his father. Fig 3D and 3E show a similar phenotype produced in both reciprocal crosses of *S*. *ruficollis* and *S*. *cinnamomea*. Some of these F1 phenotypes can not be easily distinguished from that of capuchino species observed in the wild (both males in Fig 3C), while others have not been described in wild populations (Fig 3B, 3D and 3E).

## Discussion

In this study we present the first direct evidence of hybridization among capuchino seedeater species using captive birds. We compared the viability of F1 hybrids to that of individuals obtained from conspecific pairs. Our results suggest that hatching success is lower in the conspecific pairs, whereas fledging success did not differ between conspecific and heterospecific matings. The lower hatching success in conspecific pairs may be the consequence of inbreeding in the captive birds, whereas hatching success may be higher in the hybrids when more distantly related individuals (from different species) are crossed. This pattern of decreased hatching success with increased genetic similarity has been documented before in birds [31]. The hatching success we observed in conspecific pairs is comparable to that reported for zebrafinches (*Taeniopygia guttata*, [32]) bred in captivity. Hybrid crosses show a trend towards producing excess males, potentially consistent with lower viability in F1 females (the heterogametic sex). It is also possible that F1 female hybrids show some degree of infertility. Both of these observations are consistent with the predictions made by Haldane’s rule, yet our sample size was too small to robustly conclude that F1 hybrid females are either infertile or inviable. If these trends hold true with larger sample sizes they would suggest a role for Z-linked loci in postzygotic reproductive isolation among capuchino seedeters. Campagna et al. [16] sequenced the genomes of five different species and found very low levels of genomic differentiation (mean *F*_*ST*_ = 0.008), yet 25 outlier regions or divergence peaks showed higher levels of differentiation. Importantly, 10 of the 25 peaks were located on the Z chromosome. This pattern of a disproportionate contribution of sex chromosomes to species differences (fast-Z effect) has been observed in many bird species [33].

We documented male hybrid phenotypes in capuchino seedeaters, providing information relevant to detecting hybrids in nature. We obtained four types of hybrid phenotypes. First, some hybrids showed plumage patterns that were similar to those of their parental species. Individuals with these phenotypes, if produced in nature, would not be easily identified as hybrids, suggesting that cryptic hybridization events could occur in the wild. We also obtained a male hybrid with plumage that is intermediate to that of both parental species (with pale brown plumage instead of pearly white or rufous; Fig 3B). This suggests that wild adult males with intermediate plumages could represent hybrids. A third phenotype, the product of a cross between a male *S*. *palustris* (*zelichi* morph) and a female *S*. *hypoxantha*, is very similar to that of the *urugauya* morph that has been described for *S*. *hypoxantha* (see Fig 1 and Fig 1 in [20]). It is possible that other hybrid crosses could produce a similar phenotype, and therefore we can not simply conclude that the *uruguaya* morph is the product of a *S*. *palustris* (*zelichi* morph) x *S*. *hypoxantha* cross in the wild. However, our observation supports the possibility that some of the rare intra-specific morphs could be the products of hybridization. Finally, we obtained a hybrid phenotype when crossing *S*. *cinnamomea* and *S*. *ruficollis* (in both directions) that has not been described in the wild. *S*. *cinnamomea* and *S*. *ruficollis* can be found sympatrically, thus it is possible that this hybrid either does not occur naturally or has not yet been found.

The coloration differences in male capuchino species are produced by combining a limited set of colors (e.g., rufous, tawny, etc) across a fixed set of plumage patches (e.g., crown, nape; see [34] for details). The transgressive hybrid plumage patterns produced in our study suggest that there may be complex epistatic interactions between the genes controlling coloration and patterning in the capuchinos. For example, when we crossed a species with a tawny back and a white throat and nape to one with a tawny throat and gray nape and back, we obtained a hybrid with a tawny throat, nape and back (Fig 3A). When we crossed a species with a black throat and a gray nape and back to one with a dark rufous throat, nape and back, we obtained a hybrid with a black throat, nape and back (Fig 3D and 3E). These observations suggest some colors dominate over others and that some patches may vary together in a modular way.

We conclude that F1 hybrid inviability, if present, is not strong enough in this system to be detected unambiguously in our dataset. Hybrid infertility has been observed to arise before inviability [1], and the fertility of F1 capuchino hybrids and the viability and fertility of F2 hybrids and backcrosses was not measured in this study. It remains to be determined if incompatibilities in later-generation hybrids contribute to postzygotic isolation in capuchino seedeaters. The fitness of hybrids in the wild is also unknown. The fact that capuchinos show differences almost exclusively in the coloration patterns and song of adult males suggests that prezygotic reproductive barriers are important for species integrity in this rapid radiation.

## Supporting information

S1 Table Details for the 60 crosses performed in this study. (DOCX)
