# Supplementary material for: Transgressive phenotypes and evidence of weak postzygotic isolation in F1 hybrids between closely related capuchino seedeaters
Source: PLoS One. 2018 Jun 14;13(6):e0199113. doi: 10.1371/journal.pone.0199113 (PMC6002061; doi:10.1371/journal.pone.0199113)
Supplement: S1 Table — (DOCX) [file pone.0199113.s001.docx]

Table S1. Details for the 60 crosses performed in this study. The table indicates the cross type (hybrid or pure), total number of eggs laid, total number of eggs hatched, total number of chicks fledged and their sex when known.

| **Sp. father** | **Sp. mother** | **Type of cross** | **Eggs laid** | **Eggs hatched** | **Adult total** | **Adult F1 females** | **Adult F1 males** | **Adult unknown sex** |
| --- | --- | --- | --- | --- | --- | --- | --- | --- |
| *S. minuta* | *S. minuta* | Pure | 4 | 2 | 1 | 1 | 0 | 0 |
| *S. pileata* | *S. pileata* | Pure | 6 | 4 | 2 | 2 | 0 | 0 |
| *S. cinnamomea* | *S. cinnamomea* | Pure | 2 | 0 | 0 | 0 | 0 | 0 |
| *S. cinnamomea* | *S. cinnamomea* | Pure | 5 | 1 | 0 | 0 | 0 | 0 |
| *S. cinnamomea* | *S. cinnamomea* | Pure | 4 | 2 | 1 | 0 | 1 | 0 |
| *S. cinnamomea* | *S. cinnamomea* | Pure | 3 | 2 | 0 | 0 | 0 | 0 |
| *S. cinnamomea* | *S. cinnamomea* | Pure | 3 | 2 | 1 | 0 | 1 | 0 |
| *S. cinnamomea* | *S. cinnamomea* | Pure | 3 | 2 | 1 | 1 | 0 | 0 |
| *S. cinnamomea* | *S. cinnamomea* | Pure | 2 | 0 | 0 | 0 | 0 | 0 |
| *S. cinnamomea* | *S. cinnamomea* | Pure | 2 | 2 | 1 | 0 | 1 | 0 |
| *S. cinnamomea* | *S. cinnamomea* | Pure | 4 | 0 | 0 | 0 | 0 | 0 |
| *S. cinnamomea* | *S. cinnamomea* | Pure | 6 | 2 | 1 | 1 | 0 | 0 |
| *S. cinnamomea* | *S. cinnamomea* | Pure | 5 | 2 | 1 | 0 | 0 | 1 |
| *S. cinnamomea* | *S. cinnamomea* | Pure | 8 | 7 | 1 | 1 | 0 | 0 |
| *S. cinnamomea* | *S. cinnamomea* | Pure | 3 | 1 | 0 | 0 | 0 | 0 |
| *S. cinnamomea* | *S. cinnamomea* | Pure | 5 | 3 | 1 | 0 | 0 | 1 |
| *S. ruficollis* | *S. ruficollis* | Pure | 7 | 3 | 3 | 1 | 1 | 1 |
| *S. ruficollis* | *S. ruficollis* | Pure | 2 | 1 | 0 | 0 | 0 | 0 |
| *S. ruficollis* | *S. ruficollis* | Pure | 6 | 3 | 2 | 1 | 0 | 1 |
| *S. ruficollis* | *S. ruficollis* | Pure | 2 | 1 | 1 | 0 | 1 | 0 |
| *S. ruficollis* | *S. ruficollis* | Pure | 5 | 3 | 1 | 1 | 0 | 0 |
| *S. ruficollis* | *S. ruficollis* | Pure | 5 | 3 | 0 | 0 | 0 | 0 |
| *S. ruficollis* | *S. ruficollis* | Pure | 4 | 3 | 1 | 0 | 1 | 0 |
| *S. ruficollis* | *S. ruficollis* | Pure | 2 | 1 | 1 | 0 | 0 | 1 |
| *S. ruficollis* | *S. ruficollis* | Pure | 2 | 1 | 1 | 1 | 0 | 0 |
| *S. ruficollis* | *S. ruficollis* | Pure | 2 | 0 | 0 | 0 | 0 | 0 |
| *S. palustris* | *S. palustris* | Pure | 2 | 0 | 0 | 0 | 0 | 0 |
| *S. palustris* | *S. palustris* | Pure | 3 | 3 | 3 | 0 | 2 | 1 |
| *S. palustris* | *S. palustris* | Pure | 2 | 0 | 0 | 0 | 0 | 0 |
| *S. palustris* | *S. palustris* | Pure | 5 | 4 | 3 | 1 | 1 | 1 |
| *S. palustris* | *S. palustris* | Pure | 2 | 2 | 1 | 1 | 0 | 0 |
| *S. palustris* | *S. palustris* | Pure | 2 | 0 | 0 | 0 | 0 | 0 |
| *S. palustris* | *S. palustris* | Pure | 2 | 1 | 1 | 0 | 1 | 0 |
| *S. palustris* | *S. palustris* | Pure | 5 | 4 | 1 | 1 | 0 | 0 |
| *S. palustris* | *S. palustris* | Pure | 3 | 1 | 0 | 0 | 0 | 0 |
| *S. palustris* | *S. palustris* | Pure | 3 | 0 | 0 | 0 | 0 | 0 |
| *S. hypochroma* | *S. hypochroma* | Pure | 2 | 1 | 0 | 0 | 0 | 0 |
| *S. hypochroma* | *S. hypochroma* | Pure | 2 | 0 | 0 | 0 | 0 | 0 |
| *S. pileata* | *S. pileata* | Pure | 2 | 0 | 0 | 0 | 0 | 0 |
| *S. pileata* | *S. pileata* | Pure | 3 | 0 | 0 | 0 | 0 | 0 |
| *S. palustris* (*zelichi*) | *S. hypoxantha* | Hybrid | 8 | 6 | 4 | 2 | 2 | 0 |
| *S. palustris* | *S. hypoxantha* | Hybrid | 4 | 3 | 2 | 0 | 2 | 0 |
| *S. pileata* | *S. hypochroma* | Hybrid | 5 | 5 | 3 | 2 | 1 | 0 |
| *S. cinnamomea* | *S. ruficollis* | Hybrid | 2 | 2 | 1 | 0 | 1 | 0 |
| *S. ruficollis* | *S. palustris* | Hybrid | 2 | 1 | 1 | 1 | 0 | 0 |
| *S. ruficollis* (*caraguata*) | *S. ruficollis* | Hybrid | 3 | 3 | 0 | 0 | 0 | 0 |
| *S. ruficollis* (*caraguata*) | *S. ruficollis* | Hybrid | 2 | 0 | 0 | 0 | 0 | 0 |
| *S. ruficollis* (*caraguata*) | *S. ruficollis* | Hybrid | 2 | 1 | 0 | 0 | 0 | 0 |
| *S. hypoxantha* (*uruguaya*) | *S. cinnamomea* | Hybrid | 4 | 2 | 0 | 0 | 0 | 0 |
| *S. palustris* | *S. cinnamomea* | Hybrid | 4 | 3 | 1 | 0 | 0 | 1 |
| *S. palustris* | *S. cinnamomea* | Hybrid | 2 | 1 | 0 | 0 | 0 | 0 |
| *S. palustris* | *S. cinnamomea* | Hybrid | 2 | 2 | 1 | 0 | 0 | 1 |
| *S. palustris* | *S. cinnamomea* | Hybrid | 4 | 3 | 2 | 0 | 0 | 2 |
| *S. palustris* | *S. cinnamomea* | Hybrid | 2 | 1 | 1 | 0 | 1 | 0 |
| *S. palustris* | *S. cinnamomea* | Hybrid | 5 | 2 | 1 | 0 | 0 | 1 |
| *S. palustris* | *S. cinnamomea* | Hybrid | 2 | 2 | 0 | 0 | 0 | 0 |
| *S. ruficollis* | *S. cinnamomea* | Hybrid | 2 | 0 | 0 | 0 | 0 | 0 |
| *S. ruficollis* | *S. cinnamomea* | Hybrid | 2 | 2 | 1 | 0 | 1 | 0 |
| *S. ruficollis* | *S. cinnamomea* | Hybrid | 5 | 5 | 2 | 0 | 0 | 2 |
| *S. ruficollis* | *S. cinnamomea* | Hybrid | 2 | 0 | 0 | 0 | 0 | 0 |
